# Sheldrake on Dancing

**Published:** 1829-04-01

**Authors:** 


### Sheldrake on Dancing.

Saltat Milonius ut semel icto

Accessit fervor capiti, numerusque lucernis.

*Juvenal.*

A Mr. Sheldrake has lately been adorning the " invaluable" with what he terms " lectures," de omnibus rebus et quibusdam aliis. After writing more than we mean to read on crooked feet, fencing, gymnastics, &c. the worthy " lecturer" (we should like to see his class) has kindly taken dancing under his wing, and descanted with equal learning and length on the boundless advantages which spring from the use of " the light fantastic toe." Mr. Sheldrake's definition of dancing is rather comprehensive, for " he only intends to consider it as *something* that either has, or may have, peculiar effects upon the health, as well as upon the form of those who practise it," which indeed would be equally true of a broken head, or any other mode of diversion under the sun! In the history of dancing which is given, and which is absolutely necessary, of course, in order to point out its advantages to the health of those who engage in it, we are greatly astonished, and profoundly grieved to find that the Lecturer goes no farther back than the times of Louis Quatorze. Did not the Children of Israel dance before the Golden Calf, (an example which the children of this generation are somewhat too apt to follow now)? Did not David dance before the Ark? Did not Minerva, the blue-eyed maid, dance for joy after thrashing those radical fellows the Titans, who attacked the Royal College of Divinities with *stones* even larger than the one Mr. Partridge *could not* extract? Were not Castor and Pollux Lacedæmonian dancing masters? Did not Socrates, Athena's wisest son, learn to dance of Aspasia?

Surely a discourser on dancing must have consulted the *immortal* " Essai Historique sur la Danse" of Cahusac; the work of Mercuruli, " de Saltatoriæ Effectibus,"—as well as Rousseau's Advice to Young Ladies rather to imitate the climbings and skippings of the wild goats, than the graceful steps of the Opera dancers. A lecturer indeed of Mr. Sheldrake's calibre should have mounted at least to the flood.

Although, however, the history of dancing, with what we must designate frightful indifference, is only commenced with Louis XIV. yet some highly important reflections are made for all that. Thus the Lecturer informs us, that when the ministry of the Grand Monarque, found they could not conquer Europe by arms, they determined in revenge to *beat* it in dancing, and accordingly erected a Royal Academy, in which it was taught " in the most perfect manner," and " raised to the dignity of a science." We would earnestly invite the attention of ministers, especially of his Grace of Wellington, to this point, for although he may not raise the blockade of the Dardanelles, yet think how he might silence opposition, and throw Waterloo and Vittoria into the shade by founding a Royal Academy of posture-masters, and erecting dancing into a science! In such a case Mr. Sheldrake assuredly deserves, and no doubt will obtain, for this valuable hint, the directorship, or some of the other good things in the gift of the premier. We quite envy Mr. S.

O! fortunatum nimium, sua si bona norit!

Conceiving, and religiously believing, that by proving dancing not to be injurious, he would calm the trembling solicitudes of many, and confer an " acceptable service" on mankind, Mr. Sheldrake has kindly consented to do so

" effectually, by tracing its principles to their very foundation." Now all men unfortunately, have not an opportunity of tracing the principles of dancing to their very foundation, but Mr. Sheldrake had, for he knew, nay more, was even "familiarly acquainted" with Mr. Birch, and Mr. Birch knew Madame Simonet, and Madame Simonet, and Mr. Birch, and Mr. Sheldrake between them, got to the very foundation, or as Virgil would term it, *ad ima*, of the principles of dancing, in a drawing-room belonging to one of the above illustrious individuals! For the sake of mankind, and womankind especially, we subjoin the morçeau, which Mr. Sheldrake makes so much of. Mr. S. loquitur, and Madame Simonet is the heroine of the tale.

" She successively learned to stand flat and firm upon both her feet, with her legs quite straight, and the whole of her person quite upright, but not stiff; then to lift one foot from the ground, and keep it so, for some time, without moving any part of her body; she then replaced that foot on the ground, and raised the other in the same manner. These simple actions were repeated till the pupils were quite familiar with them; they were then directed to keep the body quite erect, but not stiff, and, bearing firmly upon one leg, to raise the other from the ground, gradually and slowly, by bending the thigh at its junction with the pelvis, at the same time making the knee straight, and pointing the toe to its proper extent, but *no more*. The foot, after it had been kept in this state for some time, was returned to the ground from whence it was taken, and the other foot treated in the same manner: when quite familiarised to these actions, they were directed to walk (march, as some people will call it) slowly, performing the same motions with the feet alternately.

" The next lesson was to keep the foot turned out to a proper extent at the ancle-joint, (a circumstance which will be particularly explained in another place) to raise the foot more than is necessary in walking, keeping the toe pointed, the knee straight, but making a semicircular motion with the hip-joint, so as to turn the toe outwards, and carry it round and backwards as far as the *natural* flexion of the hip-joint will allow it to go: these motions should be made by each leg, first moving the toe forwards and round till it was carried backwards, and then throwing the foot out backwards, and bringing it gradually round, till it is placed upon the ground in the front. These may be called the elementary motions of the legs; and this mode of exercising them may, not unaptly, be called the rudiments of muscular action, as it should be used in the practice of dancing.

" When the pupils were quite familiarised to perform all the simple motions of the legs, with the greatest ease and activity, they were again directed to stand upright, with the feet close together, the body firm, erect, and motionless upon the pelvis; and, in this situation, they were directed to give every motion to the arms and to the body, without stooping, that the natural structure of the joints, and the natural actions of the muscles, would admit of. These, as in the case of the legs, were practised with every variation, till they were quite familiarised to them; they then were qualified to go to what may be called their finishing exercise; that consisted in placing themselves in any attitude they were directed to, at the instant the direction was given, and to change from that to any other, however opposite it might, to the unitiated observer, appear to be."

Now these fundamental principles of dancing are just what every dancing-master, knows much better than Mr. Sheldrake can tell him, and therefore the latter gentleman might have saved himself the trouble of spinning out so roundabout a story respecting Mr. Birch and Madame Simonet. Mr. Sheldrake has a crotchet in his head respecting what he denominates "muscular tension," and he believes that the elder Noverre and Mrs. Garrick lived, the one eighty years, the other ninety-nine, merely through "a modification" of this same muscular tension, which modification is the essence of those preparatory exercises described above, and practised by those who follow the profession of dancing! Fudge!

" It is upon the same principle only (a modification of muscular tension) that we can account for the fact that soldiers are well formed, and always healthy, *unless they are made otherwise either by accident or disease.* (!!) I have never seen a soldier with spinal curvature, or other personal deformity, or a stage dancer of either sex, with a deformed person; it is *perhaps impossible* that such things should exist, for the *plain reason* that the exercises which they begin to practise early in life, and continue regularly through its whole course, render it *impossible* for them to become so."

There is just *one other* principle besides that of a regulated modification of muscular tension, which accounts for the fact of soldiers being well-formed, (it never crossed the head of Mr. S. good easy man,) we mean, a REGULATED RECRUITING DEPARTMENT! Which of the two principles, Mr. Sheldrake's or our's, has the most to do with the matter, a corporal would very soon decide. It is not a bad idea of Mr. Sheldrake's, though certainly its novelty admits of question, viz. that soldiers are always healthy, *unless* they are made otherwise by *accident* or *disease*. Mr. Sheldrake has probably seen people (*not* soldiers) unhealthy, *without* either accident or disease! The logic of the passage we have quoted is nearly as good as the rest of the precious affair—it is impossible, quoth he, that dancing people should be ill-formed, for the plain reason that it is—impossible! certes, a very profound deduction.

After these preliminary remarks on the preliminary *steps* of the "science" of dancing, Mr. Sheldrake takes a bolder flight, and soars into the regions of the ballet, and the differences between the French and the Italian modes! He informs us that the French are better dancers than any other nation, and occupies nearly half his lecture in proving what our grandmothers knew full as well as Mr. Sheldrake. To ascribe this, however, merely to the *system* pursued in France, is downright twaddle, because when adopted elsewhere it does not and will not produce the same results. The French, as a people, are constitutionally dancers, just as the Italians are naturally musicians, and the Hollanders neither. We should like to see Mr. Sheldrake make a *figurant* of a burgomaster, notwithstanding all the preparatory manœuvres disclosed to his ravished optics by Madame Simonet, or the finest modification of regulated muscular tension invented by himself. Goldsmith, who, we readily admit, is not to be compared to Mr. Sheldrake, has observed of France—

"Alike all ages: dames of ancient days
Have led their children thro' the mirthful maze;
And the gay grandsire skill'd in gestic lore,
Has frisk'd beneath the burthen of threescore."

It is not the system that has made the French dancers—but the French, being dancers, that have made the system. The distinguished lecturer recommends, and "a gentleman, who is a very eminent teacher of dancing," fully agrees with him, that all young ladies and gentlemen should begin with the minuet, that being the very best *ground* to *go* upon. Coming from such quarters, the recommendation will no doubt be acted on forthwith throughout civilized Europe at least. In "conclusion," we are favoured with the following remarks, which, for grammatical construction, lucid description, and scientific reasoning, are perfectly unrivalled. We would not, for the universe, withhold them from our readers.

"First, the feet should *always be kept directly under the legs*, and not *to* allow them to turn or twist beyond what, for want of a better term, is called the natural form. In this state, the foot has its greatest motion directly backwards and forwards; of this motion, the greatest use is made in dancing. *It* has, likewise, a *motion in the ancle joint; it is made by turning the great toe inwards. The foot has another motion at the ancle joint; it is made by turning the toe outwards.* As much and serious injury is often sustained by persons who do not understand the structure of this joint and the proper way of managing it, to produce the effect that they desire, it will be well to explain it here.

"If a person stands upright, with his heels close together, and his toes so placed, that a perpendicular line, *passing* through the middle of the patella down the leg and foot, by the inside of the great toe, his feet will be in the strongest position it is possible for them to take; *they* will be capable of making greater exertions, and for a longer portion of time *than they can make in any other position.* This is the position in which the skaiter places his foot, when he is preparing to strike with the full power of all the muscles of his thigh, leg, foot, and toes; being prepared for this action, ***he strikes, with all his force, the inside of his great toe against the ice, and thus drives himself forward, while he stands firmly upon his other foot.***

"This action of the great toe is likewise of great importance in dancing; the dancer, indeed, often has occasion to turn his toes outwards, much more than the position that has been described; but when he does so, if he understands how he should proceed to perform it, he keeps his foot firmly in the position that has been mentioned, and turns his *leg* outwards by the *hip-joint*, which is so formed, that it is one of the strongest of the human body; *it allows of more motion, in every direction, than any other*.* We have seen one French dancer, who was so completely master of himself in this respect, that he stood *perpendicular* upon the great toe of one foot, bent his body downwards upon one side, and raised his leg on the other, and thus balanced himself in the form of a T *square*,† and in that situation turned his pirouette in a manner that was really most astonishing."

There is only one thing more astonishing than the T square of this mountebank, which is, that one person could write and another could seriously publish such rigmarole as is here transcribed. We are told, above all things, to keep our feet directly under our legs, which, considering that people in this country are not in the ordinary habit of walking on their heads seems a very reasonable and proper request. We do not therefore quarrel with this observation nor yet with the discovery that a motion in the ankle-joint is made by turning the *great toe* inwards, or the reverse, though we freely confess our utter inability to understand how moving the great toe can affect the ankle-joint at all. If we point the *toes* inwards or outwards, then the above-mentioned joint is affected, but even in this case a considerable share of the action produced depends upon inversion and eversion of the *hip-joint*, as any one may ascertain in his own person. This serves to show how much Mr. Sheldrake knows about the matter, but let it pass. We cannot for the life of us comprehend how an individual *standing* upright, with his heels close together, and "his toes so placed *that* a perpendicular line *passing* (laud we the grammar) through the middle of the patella down the leg and foot by the inside of the great toe," will be capable of making exertions both greater and longer than he could in any other position whatever.* We cannot conceive why it *should* be thus, and we know that it *is* not. An individual in such a position is really fit for nothing, unless indeed for a sawyer, and could scarcely move his upper extremities without being in danger of losing his balance. The position of firmness and stability is with one foot advanced, like the boxer, or fencer when not making his lunge. This also serves to show how much Mr. Sheldrake knows about the matter. The description of the skaiter is perfectly unique. Being fully prepared, "*he strikes with all his force*, the inside of *his great toe against the ice*" (*!!*) and thus *drives himself forward*, while he *stands firmly on his other foot*!!! We can tell Mr. Sheldrake, that the skaiter is not such an ass as to engage his great toe in any such Quixotic quarrels with the ice, nor can he by any possibility, unless it be an *Irish* possibility, be driving forward with one foot, and standing still upon the other! This latter exploit of the Lecturer's skaiter, is only surpassed by that of the "amphibious animal," at Bartholomew Fair, which the showman informs the spectators, "can't live on the land, and dies in the water."!

Seriously, since the Lectures on the

* Has the hip-joint more motion than the shoulder-joint? Mr. Sheldrake would be infinitely better employed in *attending* lectures than in *writing* them.

† The sapient lecturer will oblige us by explaining how any one could stand *perpendicularly* with his body *down* on one side and his leg cocked a-kimbo on the other! Secondly, it would give us great pleasure to learn how a T can be *square*?

* This passage is expressed with that happy degree of obscurity in which Mr. Sheldrake peculiarly excels. We find on reading it over attentively, that by placing the feet in this position, *they* (the feet) will be capable of the strongest and longest exertions! How in the name of King Log, can any human being, except Mr. Sheldrake, "stand upright" with his feet in *one position* and be making *exertions* with those same feet *at the same time*? We say again—Fudge!

"Intellectual composition of man," published in the Lancet, by the very respectable ex-president of the Bolt Court Society, we have never seen such odious trash foisted on an easily gullible public, as these precious Essays, or Lectures, or whatever we may call them. We are not amongst those who hold that ridicule is ever the test of truth, but where an author is constantly thrusting his absurd speculations into notice, and delivering nonsense ex cathedrâ, his productions become a fair mark for the most withering sarcasm the PRESS can aim. The present cacoëthes scribendi which infests all ranks, more especially the medical, like the power of the crown, "has increased, is increasing, and ought to be diminished." The list of those in the profession "who hunger and who thirst for scribbling sake," is as long as the rolls of the colleges themselves, and perfectly ridiculous to witness. Within these few years, more especially, a class of individuals, most aptly termed the "little lecturers," have sneaked into print, and issued their "fruits of dull heat and sooterkins of wit," with as much importance as if they really were good for something. We assure these gentlemen their day is run; the eyes of impartial but unsparing critics are upon them; and this novel, but slippery species of puffery will no longer do.

"Next plung'd a feeble, but a desperate pack,
With each a sickly brother at his back:
Sons of a day! just buoyant on the flood,
Then numbered with the puppies in the mud.
Ask ye their names? I could as soon disclose
The names of these blind puppies as of those.
A monumental brass this record bears,
These are, ah, no! these were the *gazetteers!*"

To return to Mr. Sheldrake; we would merely advise him to be a little more chary in future of his "Lectures." We shall keep our eye upon him and them, and furnish our readers, as occasions offer, with samples of the sort of stuff they're made off. Nothing but our sense of duty as journalists could induce us to submit to so unpleasant a task as raking up such crude and nauseous materials.

